# Psychosis and help-seeking behavior in rural KwaZulu Natal: unearthing local insights

**DOI:** 10.1186/s13033-016-0089-z

**Published:** 2016-09-20

**Authors:** Charlotte A. Labys, Ezra Susser, Jonathan K. Burns

**Affiliations:** 1 Department of Psychiatry, Nelson R. Mandela School of Medicine, University of KwaZulu-Natal, Durban, South Africa; 2 Department of Epidemiology, Mailman School of Public Health, Columbia University, New York, USA; 3 New York State Psychiatric Institute, New York, USA; 4 Institute of Health Research, University of Exeter, Exeter, UK

**Keywords:** Help-seeking, Psychosis, Stakeholders, Treatment journeys, South Africa

## Abstract

**Background:**

Growing interest in strategies regarding early intervention for psychosis has led to a parallel interest in understanding help-seeking behavior, especially in low- and middle-income countries (LMICs). Nevertheless, few LMIC studies have examined individuals with psychosis in non-urban, non-hospital settings. Using the perspective of formal and informal community service providers, we aimed to uncover descriptions of people with psychosis in a rural South African community and illuminate the potential complexities of their help-seeking journeys.

**Methods:**

We conducted a qualitative study of 40 key informant interviews and seven focus groups with stakeholders (traditional leaders, traditional healers, religious leaders, health care nurses, heads of non-governmental organizations, schoolteachers, community caregivers) in a rural Zulu community (Vulindlela). Thematic analysis of the data was performed using the inductive analysis approach.

**Results:**

Interviewees discussed 32 individuals with probable psychosis in their community and provided rich descriptions of their symptoms. A complex picture of help-seeking behavior, primarily involving informal mental health service providers, emerged. Over half of the reported cases had no contact with formal health services in the course of their help-seeking journey; while more than two-thirds never attended a hospital and only 1 in 8 accessed a psychiatric hospital.

**Conclusions:**

Our results highlight the important role of informal care providers in LMICs as well as the need for more research on mental illness and local providers in non-hospital contexts. Community stakeholders can contribute to a fuller understanding of these issues, thereby assisting in the creation of appropriate and effective mental health interventions for rural South African communities like Vulindlela.

## Background

In recent years, growing interest in strategies aimed at early intervention for individuals with incipient psychosis has led to a parallel interest in understanding help-seeking behavior, especially in low- and middle-income countries (LMICs). Within Africa, conventional approaches to investigating help-seeking behavior entail tracking either first service provider visits [1, 2] or the pathways up until hospitalization [3–7]. Most of these studies have focused on patient populations with non-specific mental disorders in urban psychiatric or general hospital settings [1–4, 6–11], with a handful involving patients visiting primary healthcare clinics [12, 13] or indigenous healers [14, 15]. Fewer studies in African contexts have focused specifically on individuals with psychosis [4, 9, 16, 17] despite its presence and seemingly unique nature in this region (e.g. in South Africa, Kark identified distinctive forms of psychotic disorders in rural KwaZulu-Natal during the 1960s [18], while Susser and Wanderling described a “non-affective acute remitting psychosis” in the 1990s [19]).

In 1991, Gater and colleagues gathered treatment path data from 11 countries using the perspective of individuals who “reached psychiatric services”; the authors themselves considered this viewpoint a limitation and called for future studies to approach this subject “from other sources of care … to complete the mapping of care providers in the communities studies” [20]. Following Gater and colleagues’ stance, one might argue that most studies on help-seeking behavior have only been able to describe partial “treatment maps” since they assume that (a) all individuals seeking care actually access formal health services and (b) hospitalization is the final destination in the treatment journey. This is erroneous in some settings, but even more problematic in a number of LMICs where formal health resources—especially in relation to mental health—are limited and often inaccessible [21–23], and many people utilize informal healthcare. Indeed, a recent systematic review of help-seeking behavior in people with mental disorders in Africa found that almost 50 % of individuals made first contact with traditional or religious healers followed by formal mental health care [24]. The authors point out that choices and decisions regarding which practitioner/s to consult are made for a host of reasons including those related to belief or preference as well as “structural health service failures.” Thus, the issue of help-seeking behavior is closely tied to the issue of causal beliefs and understandings of illness; any attempt to unravel the complexities of help-seeking behaviors should include an engagement with the beliefs that underlie and guide behavioral choices.

The development of appropriate and effective mental health services for people with psychosis in LMICs depends upon a better understanding of the place of informal community-based providers and how individuals and their families negotiate their help-seeking journeys in such settings. Citing the work of Swartz and Patel, Mkize and Uys [5] stress the importance of identifying “cultural differences at basic levels… in connection with the creation and transformation of mental health services in the new South Africa”. In a similar vein, Anderson and colleagues [25] argue the following:

More detailed studies that are designed and powered to examine ethnic differences in pathways to care are needed to elucidate the relative contributions of immigration, culture, and social inequalities… [as well as] a more detailed examination of the complex mechanisms behind this association.

They see this as being “crucial for the design and implementation of culturally sensitive and equitable mental health services.”

### Aim

With these issues in mind, and in an attempt to move beyond some of the constraints of previous research, the current study investigates local expressions of psychotic or psychotic-like phenomena and describes the help-seeking behaviors of individuals with probable psychotic symptoms (and their caregivers) through the perspective of formal and informal community service providers. A second paper focusing on explanatory models and practices of these providers will follow as the extent of interview material exceeded the scope of this article. As in a few prior studies [26–28], we included traditional healers who, as aforementioned, are key stakeholders in LMIC contexts. Also similar to preceding research of this kind (i.e. interviews, focus groups, ethnographic observations, etc.), we sought the perspectives of a broad range of community stakeholders including church leaders, non-governmental organization personnel, teachers, community clinic nurses, and health workers [14, 27, 29]. In addition to eliciting cultural idioms of severe psychological disturbance, we aimed to describe a clearer picture of what occurs in some rural South African communities characterized by limited or even absent formal health resources with the hope that this research will help illuminate the complexities of people’s help-seeking journeys.

## Methods

The current study adopted a qualitative methods approach and was conducted as part of a pilot study, *Psychotic Disorders in an African Setting: Incidence, Early Course and Treatment Pathways (FEP*-*INCET)*, designed to develop a method for screening, identification, and follow-up of individuals with incident psychosis within a rural low-income South African setting.

### Population and setting

Vulindlela is a poor rural community, 150 km west of Durban, South Africa, in a region where social inequalities and HIV rates are among the highest worldwide. The vast majority of the population of 250,000 is Zulu, with 32 % being between ages 18 and 32 years. Vulindlela has a strong traditional council with five traditional wards, each headed by a tribal chief. The tribal chiefs play a key role in community life in parallel with formal government authorities. The unemployment rate is 45 %, while more than one-third of the population age 15–34 years is HIV positive. Traditional Healers (THs) have a prominent role in health care and are clustered into groups across the 3 Vulindlela sub districts of Inadi (400 THs), Mpumuza (700 THs), and Mafunze (300 THs) respectively. There are nine primary health care (PHC) clinics across the region as well as a cadre of community caregivers (CCGs; local equivalent of community health workers)—all operated by the provincial Department of Health (DOH). For hospital care, residents are referred to the nearest town 40 km away, Pietermaritzburg, which has two district hospitals, a tertiary level general hospital, and a specialized psychiatric hospital (all under DOH). Approximately 60 non-governmental organizations (NGOs) are active in the area, representing a variety of civic interests such as youth, women, religion, politics, and community development.

### Recruitment and sample

After engaging with the traditional chief and his council who have authority in Vulindlela, the FEP-INCET research team member responsible for community liaison and project management—a bilingual Zulu psychiatric nurse—identified members of different stakeholder groups throughout the region. Our objective was to conduct semi-structured interviews with key informants that our team regarded as important sources of local knowledge, including: PHC nurses; traditional leaders (*induna)*; traditional healers (*isangoma*—diviner; *inyanga*—herbalist; *umthandazi*—faith healer); religious leaders (i.e. church ministers); heads of non-governmental organizations (NGOs); schoolteachers; and community care givers (CCGs). The goal was to gather their impressions of psychotic-like symptoms and treatment-seeking behaviors of individuals whom they assist as service providers and/or see in their communities.

The project manager met separately with the seven stakeholder groups, discussing with them the issue of mental illness (and psychosis in particular) and access to care as well as introducing them to the purpose and objectives of the FEP-INCET study. Attendees were invited to participate in either individual key informant interviews (KIIs) or focus groups (FGs) and a potential interviewee list was created with contact details. In respect of THs, the project manager used known contacts to verify individuals claiming to be THs and locate additional THs in the area (i.e. snowball sampling). Thereafter, stakeholders on the list were contacted via phone or in person and identified for availability to participate in the subsequent KIIs or FGs.

The 83 interviewees included 53 females and 30 males with an average age of 49.8 (range 25–93 years). The majority of participants were married (N = 51) and self-identified as Christian (N = 80).

### Instrument

We created and utilized a semi-structured interview guide with nine questions regarding possible symptoms seen, help-seeking behaviors of individuals with possible symptoms of mental illness and their caregivers in that community, experience of referrals and impressions of treatment outcomes, personal beliefs about causes of mental illness, and community behavior/attitudes towards individuals with mental illness [30]. We aimed to formulate questions that would have as little influence as possible on respondents’ answers; for example, “do you see individuals with *ukuphazamiseka ngokomqondo* [Zulu word for “disturbance of the mind”, a term agreed upon by the Zulu speakers of our research team] in your community? If so, please describe the types of disturbances you see.”

### Procedures

KIIs and FGs were held at various locations in Vulindlela: community/municipal halls, NGO facilities, primary health care clinics, private homes of traditional healers, and schools (primary and secondary). Forty individual KIIs (comprised of 10 THs, 5 members of NGOs, 5 PHC nurses, 5 CCGs, 5 teachers/principals, 4 religious leaders, and 6 traditional leaders) and seven FGs (consisting of 6–7 participants from each stakeholder group—i.e. a traditional healer group, a PHC nurse group, etc.—totaling 43 participants) were conducted with no overlap in participants from KIIs and FGs.

Interviews took place between April and June 2013 with each individual interview (KII) lasting from 60 to 90 min, and each focus group (FG) from 90 to 120 min. An English-speaking clinical psychologist (CAL), with the project manager acting as a simultaneous English-Zulu interpreter, conducted the interviews, some of which were in English and others in a mixture of Zulu and English as interviewees were asked to choose their language of preference. All interviews were recorded digitally with a hand-held device; in addition, a bilingual psychology master’s student recorded notes on verbal and non-verbal interactions of participants, the interviewer, and the interpreter. All recorded interviews were transcribed and translated into English.

### Data analysis

#### Coding of material

Interviews were coded by CAL using Atlas.ti software [31] and analyzed according to the main themes that emerged from the data. Using the inductive analysis method [32], CAL developed categories and subcategories for psychological conditions, behaviors, treatment type, provider locations, and sequence of treatment. Throughout this process, CAL and JKB regularly reviewed interview material together, reorganizing codes accordingly.

#### Identification of likely psychotic symptoms/illness related by participants

During the interviews, community service providers described numerous individuals whom they had encountered and perceived as exhibiting symptoms suggestive of psychosis. Some of these were individuals who personally (or their families/caregivers) had approached stakeholders seeking assistance for their mental health problems; others were community members or relatives of stakeholders.

By consensus review and discussion of the interviewees’ detailed descriptions, two of the team’s investigators (CAL and JKB) reached agreement on which individuals described by participants were likely to have been exhibiting signs of a psychotic illness. Symptoms and behaviors identified as probably being psychotic in nature (e.g. seeing invisible people from the Bible, repeated checking to see if people are listening in, etc.) were grouped into “psychotic symptom/behavioral” (PSB) clusters (see examples under Results below). Cases involving reported comorbid substance use were removed.

Finally, using interviewee reports, CAL identified help-seeking behaviors of the individuals with likely psychotic illness.

## Results

### Psychotic symptoms/behaviors

Thirty-two individuals with likely psychotic illness were identified from descriptions provided by interview participants. Review of key informant and focus group interview material pertaining to these 32 “cases” yielded 22 “psychotic symptom/behavioral clusters” (PSB) clusters.

The most commonly described PSBs were: “aggression/agitation”, “called ‘crazy’”, “strange behavior”, “auditory hallucinations”, “confusion”, “poor hygiene”, and “talking nonsense.” Each cluster contained several descriptions of symptoms and/or behaviors that were judged to refer to the same phenomenon. For instance, the PSB cluster “strange behavior” included: walking around naked outside, undressing oneself in public, picking up papers, sitting outside other people’s houses at night, sleeping on the grave of one’s murder victim, making peculiar comments, wearing children’s clothes, and only wanting to eat raw meat. Table 1 lists the PSB clusters and gives examples of specific symptoms and behaviors comprising these clusters.

**Table 1:** Psychotic symptom/behavioral (PSB) clusters and specific symptoms/behaviors

Symptom/behavioral clusters	Specific symptoms and behaviors
Aggressive/agitated	Aggressive in body language; aggressive verbally (threatens; insults others; calls out people’s names; shouts at others); extreme aggression Anger: talks angrily; angry body language Violence: throws objects (e.g. food); tears up objects; bangs wall with fists and head; fights; beats wife; murder Causes trouble Change in behavior: rude, talks disrespectfully, uses vulgar language Induces fear in others Screams; shouts throughout the night Treatment noncompliance (traditional and Western-like) Demands money; demands treatment Hysterical Irritable Uncooperative and needing supervision (e.g. refusing to eat; refusing to bathe)
Called “crazy”	Acts “nuts”, mentally disturbed (temporary), mentally ill (chronic), very sick mentally, mentally affected, mentally confused, went “crazy”, looks like a “mad person”, “fully disturbed in such a way that everything went upside down during that day” Driven “mad” by ancestral spirits; “lost his mind” after cultural ritual
Strange behavior	Hoards rubbish Picks up paper Makes peculiar comments Writes inappropriate things Undresses in public; walks around naked or partially naked in public; walks barefoot; sleeps on grave of his murder victim; goes out in the rain; only wants to eat raw meat; sits outside people’s houses at night as if he wants to get inside; sings traditional songs never taught to him after cultural ritual; takes others’ washing from clothesline; takes children’s clothes and wears them; spits saliva, calls the name of the late radio announcer and then climbs up inside the house Involved in repeated car accidents (smashed new cars)
Auditory hallucinations	Hears voices (i.e. grandfather, etc.); hears voices after cultural ritual Says things aloud as if he is talking to visible people Talks to self; talks to self as if talking to others Asks if you hear the people he/she hears Hears voices to kill self by going into road or hanging self (COMMAND)
Confusion	Shows signs of mental confusion (e.g. unable to recognize photos of wife and children) Disappears from home/community Gets lost Disoriented (e.g. not knowing day’s date)
Poor hygiene	General self-neglect Not washing Rotten teeth Wears torn clothes Wears dirty clothes
Talking nonsense	Answers inappropriately Talks in a way that shows he is “unwell” Irrelevant talking; says nonsense; says incomprehensible things Says untrue things
Sleep problems	Not sleeping at night Lack of sleep Restless Sleeps in street and outside in veld
Scared	Appears frightened as if he sees something harmful Looks around frightened as if someone will listen to conversation Fears everything including a “bang” sound Traumatized
Called “psychotic”	Psychotic behavior Appears to be in a psychotic state
Social/occupational dysfunction	Stopped working Left job Stopped attending school
Wandering	Wanders back and forth on road Wanders from house to house
Visual hallucinations	Appears to see something that is not there; says people who others do not see are passing by Describes seeing invisible people from Bible Says saw invisible people after cultural ritual Says saw a snake entwining itself on bus steering wheel while driving
Psychomotor problems	Reports blurred vision/being blind Unable to walk Feels physically sick; feels like vomiting
Weight change	Not eating; does not eat when mentally sick
Social withdrawal	Avoids social interaction Avoids eye contact/looks down Does not want to be touched
Paranoia	Checks to see if people are listening Looks around in a paranoid way Says there are people who want to hurt him Answers as if someone is attacking him and out to get him
Hypersexuality	Asks women to move around in sexual way so he can get aroused Tells women he yearns for them Wants to rape women
Memory problems	Concentration difficulty Reports no memory of behavior during acute illness
Lack of insight	Lacks insight about being mentally ill
Delusions of grandeur	Says father is Nelson Mandela Says whole earth belongs to him and all these other people pay him rent
Not communicating	Does not answer questions

The following extracts from interviews provide some illustrations of the abovementioned psychotic symptoms and behaviors (e.g. hallucinations; talking nonsense; wandering; etc.):

*Well, there is one person who is mentally disturbed.*

*One sort of like gets frightened as if there is something he sees.*

*This person’s… while talking is saying there are people who want maybe…*

*want to do something to him, but you don’t see those people.*

*Sometimes he asks you: “do you hear these people?”*

*And then he says, “They go past!”*

*He says he sees Gorrah and Goriat.*

*“Here they go past. They want me.”*

*He wants you to help him… and look for Gorrah and Goriat and listen to what they’re saying. He wants help.*

*He is a gentleman… it’s someone I’ve been thinking is fine because when he comes across you can’t tell that he’s mentally ill.*

*You only… when you start talking.*

*This… it doesn’t happen continuously.*

*This has been going on for three months now.*

*But he does not have insight right now that he is disturbed as he is now talking about Gorrah and Goriat.*

*They have just gone somewhere because… now it’s a problem.*

*He has even stopped working. These days he is not even going to work.*

- Case 2 as described by KII ccg 2

*One example: one man was involved in a car… in a bus accident.*

*He saw a snake twining itself whilst he was driving a busload on his steering wheel. Then it was… they say it was such a big snake*—*colorful.*

*And it put his head face to face with him.*

*And he just screamed and left the bus… to move on his own*

*and he lost his mind from that day.*

- Case 9 as described by KII ngo 5

*There is another female… she doesn’t wash herself…*

*And talks, he [she] is, you know, confusion… what he’s [she’s] telling…*

*he [she] talks nonsense or something like that.*

*She does talk to other people.*

*So she says things that aren’t true.*

*It’s a girl with rotten teeth. She doesn’t wash herself.*

*Sometimes she goes barefooted.*

*Wandering around… going from house to house.*

*She tells people that they were beating her at home, they’ve stolen her money, telling lies.*

- Case 1 as described by KII ccg 1

*The one person in my neighborhood who was mentally disturbed…*

*was deserted by her husband… because she was… infected with HIV*

*And then she was hurt.*

*Yes, it was during that time when she became confused and eventually left the job… her job.*

*She shouts… He [she] talks about things that are not well understood.*

*Ja, it’s the speech that does not make sense, but the words are normal words.*

*When you talk to her, she answers things that are not appropriate…*

*She appears frightened sometimes as if she’s afraid.*

- Case 3 as described by KII ccg 3

*I (Tr.): I may say it is here because I know it has happened maybe to people close to me in some years.*

*His parents passed away and left 20 cows but he did not do any job and did nothing.*

*They all died.*

*After they died, he went crazy and picked up papers.*

- Case 5 as described by KII local leader 6

*I (Tr.): Like my neighbor: he walks partially naked, what can I say.*

*He walks and talks to girls, and does funny things… like telling them he yearns for them, let me put it that way.*

*He does not dress; maybe he wears underwear and walks barefeet and does anything…*

*He talks loudly and we can hear him.*

*And sometimes he talks to himself as if he is talking to people and he says “You so and so… this and that.”*

*He does not bathe; he is dirty.*

- Case 31 as described by FG ngo 7

### Help-seeking behaviors

Descriptions of help-seeking behavior in the 32 individuals described by stakeholder participants and likely to have psychotic illness revealed complex and varied choices in this community. The majority of described individuals consulted traditional healers as their first point of contact. Primary care clinics and community resources such as churches and community caregivers were less frequently chosen as first point of contact, while very few individuals commenced their help-seeking at a hospital. Other first points of contact sought for help included home, police, school, and local traditional leader.

Interview participants gave examples of many different and varied patterns of help-seeking behavior. In approximately half of the individuals, only one service provider was consulted; while roughly a third visited two providers and a fifth consulted three or more providers. Table 2 shows the patterns of help-seeking behavior for each of the described individuals including the categories of service provider reportedly consulted.

**Table 2:** Patterns of help-seeking behavior of suspected psychotic individuals (“cases”) described by participants (n=32)

Individual (“Case”) ID number	1st step	2nd step	3rd step	4th step	5th step
3	CCG	Medical (General hospital)	Social worker		
25	CCG	Medical (Clinic)			
1	CCG	Medical (Mobile clinic)			
18	Church				
21	Medical (Clinic)	Medical (Psychiatric hospital)			
15	Medical (Clinic)	Tent church	Home cultural ritual	Medical (Psychiatric hospital)	
13	Medical (Clinic)	Medical (Hospital—unspecified)	Medical (Clinic)		
20, 26	Medical (Clinic)				
30	Trad healer (Faith healer)	Medical (Hospital—unspecified)	Trad healer (Faith healer)		
29	Trad healer (Faith healer)				
4	Medical (General hospital)	CCG	Medical (Clinic)		
12	Home	Traditional healer			
2	Home prayer	Trad healer (Sangoma^a^)			
11, 27, 31	Trad healer (Inyanga^b^)				
28	Local traditional leader	Unspecified location for pension grant			
14	Police	Medical (Clinic)			
24	Police	Medical (Psychiatric Hospital)	Medical (General hospital)		
32	Medical (Psychiatric hospital)	Medical (Other psychiatric hospital)	Medical (General hospital)	Trad healer (Sangoma^a^)	Trad healer (Inyanga^b^)
19	Religious leader				
6	Religious leader’s wife				
7	Trad healer (Sangoma^a^)	Home cultural ritual	Sangoma^a^		
10	Trad healer (Sangoma^a^ and Inyanga^b^)	Medical (General hospital)			
16, 22, 23	Trad healer (Sangoma^a^)				
8	School	Remedial specialist teacher	Home cultural ritual		
17	Traditional healer	Home cultural ritual			
5	Traditional healer				
9	Witchdoctor	Medical (General hospital)			

*CCG* community care giver

^a^Sangoma—traditional healer who is a diviner

^b^Inyanga—traditional healer who is an herbalist

There were some reports of parallel use of services, specifically a mix of traditional and modern treatments at the same time. For example, a visit to a psychiatric hospital, a second psychiatric hospital, general hospital, sangoma, and finally inyanga; or sangoma and inyanga at the same time followed by a general hospital; or clinic, tent church, home (cultural ritual), and then a psychiatric hospital. The following extracts from interviews illustrate some of these help-seeking behaviors of the 32 “cases” suspected of psychosis, as related by interview participants:

*And people said [it was] because he did not want to perform the ritual [which] his father [had] said: “when I die, you must slaughter a cow. And you must do it. You must come back from where you are working and slaughter that cow for me.”*

*And he didn’t come back. He didn’t slaughter the cow.*

*Now his father came in the form of a snake… and was angry with him.*

*The witchdoctors tried to help him…. they couldn’t.*

*The hospitals tried to help him…. they couldn’t.*

*Up to this day, he walks the streets.*

*That’s why I’m confirming that at times it can be the… the neglecting (of your) ancestors.*

- Case 9 as described by KII ngo 5

*Yes it’s a girl. So with the help of tablets, being taken to Z [psychiatric hospital], Y [psychiatric hospital], X [general hospital]… there was no change.*

*They took her to izinyanga; there still was no change.*

- Case 32 as described by FG ngo 7

Notably, fifty percent of the 32 individuals had contact with traditional healers at some point during their help-seeking journey. Also, only 46.9 % had contact with formal health services (i.e. primary care/mobile clinics and general/psychiatric hospitals): just over a quarter (28.1 %) of the “cases” attended community clinics, less than one-third (31.3 %) went to hospitals (and only 12.5 % to psychiatric hospitals), and 12.5 % used both clinics and hospitals. We add a caveat that these percentages are not meant to convey accuracy but rather the raw numbers reported by interviewees.

Further interview extracts illustrate this complexity in help-seeking behavior; some participants reported the sole use of traditional treatments:

*Yeah, yeah. I remember my uncle… my mother’s brother…*

*he just, he just got disturbed.*

*Okay, he was talking to himself whatever, hearing voices.*

*And then because my grandfather was from that side X… a rural area, yeah. X. And then… he [grandfather] didn’t want to take him to hospital.*

*He just take him straight to X [geographical area]…*

*because that’s where my grandfather was born.*

*It was an inyanga.*

*He just packed their things and go there and stay there.*

*When they came, back, we didn’t know what happened there…*

*but he was normal ‘til today…*

*Because never went to the clinic. He never went to hospital. He never contacted any doctor.*

- Case 11 as described by KII nurse 4

*I (Tr.): In my neighborhood, somebody killed another person. The family tried to take him to a certain inyanga to take away his lust to kill people [through cleansing]. [But there’s been no difference].*

- Case 27 as described by FG nurse 3

*And then he was taken to a traditional healer and was told that he did not do things right: “You must do this ritual… because you did your father’s things because you took your father’s things and you did not do a Zulu cultural tradition.”*

*…. He has slaughtered all the cows or sold all 20 of them; and he was now only left with craziness.*

*… he is still like this even after he has consulted the traditional healers. They told him that his craziness was caused by 1 and 2. If you can do this, you will succeed. He cannot do it because he does not have anything to use towards this.*

- Case 5 as described by KII local leader 6

Other participants described the use of formal health services alone with reportedly positive and negative outcomes:

*I (Tr.): The relatives took him to where he could get help.*

*They took him to the clinic.*

*There was a change according to the family; he was better.*

*I heard that he was given tablets and then he was on this treatment at home, you know.*

- Case 21 as described by KII religious leader 4

*It has happened within my family: my elder sister’s son who was taken from J being mentally disturbed…*

*They tried taking him to Z [psychiatric hospital] but they couldn’t help;*

*then they went to X [general hospital]… that didn’t help.*

*Until he ran away from the hospital and was knocked by a car and was found dead.*

- Case 24 as described by KII traditional healer 4

## Discussion

### Local descriptions of psychotic illness

Stakeholder participants in our study described numerous individuals they had encountered exhibiting symptoms and behaviors of what they regarded as mental illness. Among these descriptions were individuals we, by consensus discussion, considered were likely to have manifested psychotic symptoms and psychotic illness. Accounts of these symptoms were varied and typically the content (e.g. of delusions) was related to the local context (e.g. saying his father was Nelson Mandela).

Careful consideration of these symptoms (and the symptom/behavioral clusters into which we grouped reported symptoms) reveals marked similarities to recognized biomedical features of *psychosis*, for example: hallucinations (auditory, visual); delusions (delusions, paranoia, being scared); disorganized thoughts and speech (talking nonsense, confusion); disorganized behavior (aggressive/agitated, strange behavior, wandering, psychomotor problems, hypersexuality); negative symptoms (poor hygiene, social withdrawal, not communicating); cognitive symptoms (lack of insight, memory problems), vegetative symptoms (sleep problems, weight change) and social and occupational dysfunction.

This finding is not unusual as it supports the research of other authors who have shown that, within the African context, community-based stakeholders (including lay people and formal/informal care providers) do recognize what appear to be core psychotic symptoms and behaviors as indicative of severe mental illness. Ventevogel and colleagues (2013), for example, report descriptions of *moul* (fighting, walking around naked, collecting rubbish) and *mamali* (throwing stones, talking when no one is present, bad hygiene, social isolation) from South Sudan; *erisire* (beating people, walking naked and aimlessly, talking about things that are not relevant, not realizing they are ill) from the Democratic Republic of Congo; and *ibisazi* (aggression, going naked, collecting useless things, neglecting personal hygiene) from Burundi [27]. Likewise, Sorsdahl and others [28] interviewed traditional healers in Mpumalanga Province in South Africa, who described the following as characteristic of severe mental illness: “violence, picking up garbage, talking randomly, walking for long periods of time and undressing in public”. Other stakeholder descriptions of psychosis include *eddalu* or *ilalu* from Uganda [14], “thought insertion and thought control by jinn’s or witches” from Mali [33], and *marata* (“mind is turned inside out,” “throws off his clothes and walks naked”) from the Borana nomads in Ethiopia [34]. Finally, from their research with traditional healers in Nairobi, Kenya, Mbwayo et al. [35] concluded that “traditional healers were able to recognize certain mental disorders with psychosis being the most easily recognized”.

### Help-seeking behavior for psychosis

In their systematic review of the role of traditional healers in the help-seeking experiences of people with mental disorders in Africa, Burns and Tomita [24] argue that “in contexts such as these, where formal health resources are often scarce and/or inaccessible, it is likely that a substantial portion of individuals requiring or seeking care for mental health disorders never in fact reach formal health services.” In the current study, based on descriptions provided by interviewees of the help-seeking behavior of individuals with psychosis and their caregivers, there is strong support for this statement. Nearly half of the individuals described in our study never made contact with formal health services in the course of their help-seeking journeys; while more than two-thirds never attended a hospital. In contrast, 50 % of cases consulted traditional/faith healers at some point in their help-seeking journeys, which is similar to the findings of a meta-analysis [24]. The fact that only 1 in 8 accessed a psychiatric hospital supports the commonly made assumption in LMICs that many people with mental disorders, including severe mental illnesses [36], do not receive formal biomedical treatment. It also highlights the point made by several previous authors (e.g. [20]) that research on help-seeking behaviors which confines itself to clinical populations of patients in hospitals and clinics is significantly limited and might only capture partial “treatment maps” of those communities (i.e. might exclude locally available services, like traditional healers, religious leaders, etc.).

Our findings provide a glimpse into what might be missing from these “treatment maps” by describing some of the help-seeking behavior and journeys of those individuals not accessing formal biomedical care. Of the 17 “cases” described in our study who never consulted formal health services of any kind, the majority (15) made use of solely traditional and religious service providers in their help-seeking journeys, confirming that these informal providers constitute a hugely important community-based resource for people with severe mental illness in a number of low-income contexts [37]. Specifically, the help-seeking behaviors of these individuals in Vulindlela included a combination of consultation with a traditional healer or religious person and the performance of certain rituals at home, sometimes followed by a return to a healer. This suggests that healers incorporate their clients’ family and home environment into the interventions they initiate in managing their clients’ distress—an approach that is well described in literature documenting the healing practices of traditional and religious providers [28, 38].

An additional insight from our results is that making contact with formal health services (and psychiatric services in particular) does not imply that individuals with psychotic symptoms have reached the endpoint of their treatment journeys. In the case of two individuals described by stakeholders, contact with hospitals (ID 30: unspecified hospital and ID 32: psychiatric hospital) was followed by contact with traditional (faith; sangoma, then inyanga) healers. It would be purely speculative to draw conclusions as to why these individuals sought further care from these informal providers, but possibilities include a lack of satisfaction with biomedical services and/or a decision to draw upon both or even multiple forms of care and the varied interventions they offer.

Perhaps the most striking finding from this study is the extraordinary variety and complexity of help-seeking behavior described by participants, an observation made by several previous authors [13, 20]. Overall, by the time their cases were described, 32 individuals had pursued 27 different treatment paths. No clear pattern of help seeking was apparent. Rather surprisingly, half had made contact with only one provider to date, in most cases either a religious or traditional practitioner. With the proviso that the point at which our research was conducted would not necessarily mark the endpoint of these individuals’ “journeys” and that further help-seeking could have followed subsequent to our interviews, this raises the question of whether the first (and only) provider was able to meet these individuals’ mental health needs, thereby negating the need for further help seeking? Or alternatively that, even without treatment, the natural course of the illness might have resulted in spontaneous remission as seen in the cases of acute remitting psychosis during the aforementioned World Health Organization (WHO) Determinants of Outcome Study [19] and as noted in Nortje et al.’s recent systematic review [39]? Clearly, we need to gain a deeper understanding of treatments offered for psychosis and other mental disorders by informal practitioners, the outcomes of these treatments, and the relative efficacy of these practitioners in respect of different mental disorders. This should be an important objective in future research aimed at improving treatment and support for individuals with psychosis within low-income settings.

### Informal providers—their “scope of practice” and effectiveness in respect of psychosis

There is a common perception that in the African context traditional and faith healers more typically treat individuals with common mental disorders, rather than psychotic disorders [13, 40]. There is however some evidence that what might be termed healers’ “scope of practice” does include treatment of individuals with psychosis [15, 39, 41]. For example, Abbo and colleagues [41] found that of the 233 clients with current mental disorders they evaluated at traditional healers’ practices in Eastern Uganda, 29.7 % had a psychotic disorder; while Sorketti et al. [15] found that the proportion with psychosis among 405 patients with mental illness admitted in traditional healing centers in Sudan was 34.6 %. In both cases, psychosis was the most common clinical diagnosis using the Mini International Neuropsychiatry Interview (MINI).

While the focus of this paper is not to interrogate the question of whether informal healers are effective at treating psychosis (e.g. [39, 42]), it is notable that in some of the cases described in our interviews, stakeholders expressed the opinion that “patients” had recovered following treatment by traditional or faith healers. Within the sub-Saharan African context, there has been only one diagnostically rigorous study to date describing treatment outcome for psychosis by traditional healers [38]. In Uganda, Abbo and colleagues evaluated clients of traditional healers at baseline and again at 3 and 6 months into treatment; more than 80 % of these individuals received concurrent traditional healing and biomedical services. The authors reported significant reductions in psychotic symptoms and improvement in functioning, thereby suggesting benefits of combining treatment systems for patients with psychosis. While encouraging, this concept of collaboration obviously requires further research (as recommended by Gureje et al. [43]) in different contexts and regions of the continent and, where possible, the application of more rigorous methodologies that can demonstrate causal relationships between specific interventions and defined outcomes.

### Limitations

There are several limitations that need to be acknowledged in our study, which should caution against overinterpreting or overemphasizing the significance of our findings. Firstly, our method of identifying “cases” of community-based individuals with psychotic-like symptoms and behavior through stakeholders’ descriptions in interviews, means our data is in effect second-party data. While such an approach could arguably reduce the objectivity and validity of the data (e.g. omitting other issues like dissociation), we believe the benefits of this novel approach outweigh these limitations as it allows us to access otherwise unobtainable information. As Rasmussen and colleagues [44] put it, the approach allows for a focus on “interpretation by cultural insiders”; in the case of the current study, it allows us to gain insights into the help-seeking behaviors and treatment journeys of people who are largely “operating” outside of (or in parallel with) the formal health system.

A further limitation lies in the fact that we did not include as interviewees other potential local stakeholders who may also play an important role in these help-seeking journeys, such as police, social workers, family, friends, and of course, patients themselves. Previous help-seeking behavior research has confirmed that individuals with mental health problems frequently make contact with these other role players [20, 29, 45].

Other limitations of our research include the absence of information on: delays experienced and duration of symptoms before accessing care, patient satisfaction with the various forms of care provided, subsequent help-seeking of reported cases (i.e. receiving additional services post-interview), and measures of the effectiveness and outcomes of such interventions.

### Future research

Attention to local knowledge is fundamental when discussing or thinking about how individuals with mental health needs access care in LMICs, particularly within cultural traditions that are distinct from “Western” systems. Local knowledge provides us with insights into local expressions of mental illness and into the lived experiences and journeys of care of patients, their families, and caregivers. It also sheds light on what can happen to individuals after they have contact with formal health services, which is equally important for ongoing treatment compliance, treatment overlap, and recovery outcomes. As we have seen in this study, the hospital or clinic is not always part of the treatment journey or the endpoint of healing for individuals with psychotic illness. Further research that is located outside of the formal health system will provide a more complete picture of mental illnesses, their prevalence, and their treaters in communities like Vulindela.

## Conclusions

Our study highlights the important role played by informal healers and providers in contexts where biomedical services are scarce and/or inaccessible [21–23]. Within such settings, building collaboration between formal and informal providers may be a key strategy in improving early detection, treatment, and aftercare of psychosis and other severe mental illnesses [24], provided more is known about the appropriateness of particular interventions [46]. If we are to ensure that services for people with these illnesses are “culturally sensitive and equitable” [25] and above all effective, we need to focus research efforts on exploring help-seeking behavior related to mental distress and illness outside of the usual scope and gaining a fuller understanding of how the various providers encountered in these journeys can best contribute to recovery.

## Abbreviations

CCG: community caregiver
DOH: Department of Health
FG: focus group
FEP-INCET: First Episode Psychotic Disorders and Incident Rate, Early Course and Treatment Pathways
HIV: human immunodeficiency virus
KII: key informant interview
LMICs: low- and middle-income countries
MINI: Mini International Neuropsychiatry Interview
NGO: non-governmental organization
PHC: primary health care
PSB: psychotic symptom/behavioral
TH: traditional healer
WHO: World Health Organization
